# Down-Regulation of the Oncogene PTTG1 via the KLF6 Tumor Suppressor during Induction of Myeloid Differentiation

**DOI:** 10.1371/journal.pone.0071282

**Published:** 2013-08-16

**Authors:** Pei-Yi Chen, Jui-Hung Yen, Ruey-Ho Kao, Ji-Hshiung Chen

**Affiliations:** 1 Institute of Medical Science, Tzu Chi University, Hualien, Taiwan; 2 Center of Medical Genetics, Buddhist Tzu Chi General Hospital, Hualien, Taiwan; 3 Department of Molecular Biology and Human Genetics, Tzu Chi University, Hualien, Taiwan; 4 Department of Hematology-Oncology, Buddhist Tzu Chi General Hospital, Hualien, Taiwan; Ohio State University Medical Center, United States of America

## Abstract

The aberrant expression of proto-oncogenes is involved in processes that are responsible for cellular proliferation and the inhibition of myeloid differentiation in acute myeloid leukemia (AML). Pituitary Tumor-Transforming gene 1 (PTTG1), an oncogenic transcription factor, is abundantly expressed in various human cancers and hematopoietic malignancies. However, its expression in normal leukocytes and most normal tissues is very low or undetectable. The mechanism by which PTTG1 overexpression modifies myeloid cell development and promotes leukemogenesis remain unclear. To investigate the mechanistic links between PTTG1 overexpression and leukemia cell differentiation, we utilized phorbol 12-myristate 13-acetate (PMA), a well-known agent that triggers monocyte/macrophage differentiation, to analyze the expression patterns of PTTG1 in PMA-induced myeloid differentiation. We found that PTTG1 is down-regulated at the transcriptional level in PMA-treated HL-60 and THP1 cells. In addition, we identified a binding site for a tumor suppressor protein, Kruppel-like factor 6 (KLF6), in the PTTG1 promoter. We found that KLF6 could directly bind and repress PTTG1 expression. In HL-60 and THP1 cells, KLF6 mRNA and protein levels are up-regulated with a concordant reduction of PTTG1 expression upon treatment with PMA. Furthermore, KLF6 knockdown by shRNA abolished the suppression of PTTG1 and reduced the activation of the differentiation marker CD11b in PMA-primed cells. The protein kinase C (PKC) inhibitor and the MAPK/ERK kinase (MEK) inhibitor significantly blocked the potentiation of PMA-mediated KLF6 induction and the down-regulation of PTTG1, indicating that PTTG1 is suppressed via the activation of PKC/ERK/KLF6 pathway. Our findings suggest that drugs that increase the KLF6 inhibition of PTTG1 may have a therapeutic application in AML treatment strategies.

## Introduction

Acute myeloid leukemia (AML) is a hematologic disease characterized by genetic mutations that enhance the proliferative activity of blood cells and affect their ability to differentiate and undergo apoptosis. As AML is characterized by the inhibition of myeloid differentiation, a number of studies have investigated the role of myeloid-related transcription factors in this disease. Transcription factors play important roles during hematopoiesis, from stem cell maintenance to lineage commitment and cell maturation. The inappropriate expression of these factors may subvert normal programs of cell proliferation, differentiation and survival. Activation of transcription factors such as AML1, CREB, RARα and MLL is involved in acute leukemia [1], [2], [3]. However, a much larger number of genetic lesions, including the aberrant expression of proto-oncogenes and disruption of tumor suppressor genes, are most likely involved in the processes responsible for the acquisition of the phenotype of leukemia cells. Identifying these genes and analyzing their functions can not only provide fundamental insights into normal physiology of leukocytes but can also advance our understanding of how the deregulation of networks contributes to leukemogenesis.

Pituitary Tumor-Transforming gene 1 (PTTG1), an oncogenic transcription factor, was originally isolated from rat pituitary tumor cells [4]. PTTG1 is abundantly expressed in most invasive tumors and hematopoietic malignancies. However, its expression in normal leukocytes and most normal tissues is very low or undetectable [5], [6], [7], [8]. Structural homology analysis indicated PTTG1 is a vertebrate securin that regulates sister chromatid separation [9]. PTTG1 functions in remarkably diverse processes including mitosis, DNA repair [10], gene regulation [11], organ development and metabolism [12], [13]. The overexpression of PTTG1 induced cell transformation *in vitro* and tumor formation in nude mice [9], [14], [15]. The overexpression of PTTG1 has also been demonstrated to promote cell proliferation, tumor metastasis and invasiveness [16], [17], [18]. Thus, PTTG1 may represent a molecular marker of or a potential therapeutic target for many cancers [17], [19].

The overexpression of PTTG1 has been found in several poorly differentiated leukemia cell lines (i.e., HL-60, K-562, MOLT-4 and Raji) and lymphoid neoplasias [20], [21]. However, little is known about the exact functions of PTTG1 in cellular differentiation. Recent studies showed that PTTG1 expression inhibits the differentiation of adipocytes [22]. In keratinocytes, PTTG1 can alter the proliferation status by modulating the expression levels of regulatory proteins in the G2/M phase transition and excess PTTG1 primarily suppresses the early differentiation of keratinocytes [23]. Furthermore, mice that are deficient in the *Pttg* gene exhibit thrombocytopenia and a decreased proliferation of bone marrow stem cells (BMSCs) [12], [24]. These observations suggest that PTTG1 may have a dual role in promoting cell proliferation and suppressing cell differentiation.

The mechanism by which PTTG1 overexpression modifies myeloid cell development and promotes leukemogenesis remains unclear. In this study, we sought to determine the mechanisms and signaling pathways linking the gene regulation of PTTG1 to myeloid cell differentiation. We used phorbol 12-myristate 13-acetate (PMA), a well-known differentiation agent that triggers monocyte/macrophage differentiation, to analyze the expression patterns of PTTG1 in PMA-induced myeloid differentiation. We found that PTTG1 is down-regulated at the transcription level during the differentiation process by the tumor suppressor KLF6. We found that upon PMA treatment, KLF6 is activated via the protein kinase C (PKC)/ERK pathways. Taken together, to our knowledge, this is the first study to identify the negative regulatory mechanism of PTTG1 gene expression in hematopoietic cells. Our results suggest that KLF6 suppresses the PTTG1 function to induce myeloid cell differentiation.

## Results

### PTTG1 is Overexpressed in the Undifferentiated Leukemia Cell Lines

**Figure 1:**
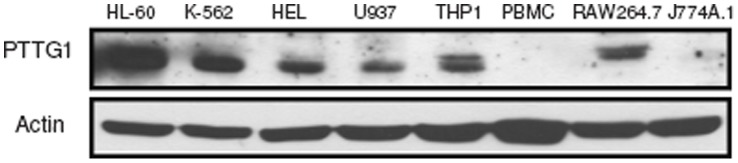
PTTG1 is up-regulated in human leukemia cells. Western blot analysis was performed to detect PTTG1 and actin proteins in human leukemia cell lines HL-60, K-562, HEL92.1.7, U937, THP1, PBMC (peripheral blood mononuclear cells) and mouse macrophage RAW264.7 and J774A.1 cells. Mouse J774A.1 cells were more differentiated, compared with the five less differentiated human leukemia cells. PBMC was used as a normal cell control.

It has been reported that PTTG1 is overexpressed in samples from patients with hematopoietic neoplasms or myelodysplastic syndromes [5]. To test whether PTTG1 proteins are over-expressed in leukemia cells, the level of PTTG1 protein in cultured leukemia cell lines was measured by Western blot analysis. As shown in **Figure 1**, abundant levels of the PTTG1 protein were detected in leukemia cell lines, including HL-60, K-562, HEL, U937 and THP1 cells. However, the PTTG1 protein was expressed lower in mouse monocyte/macrophage RAW264.7 cell line, and was not detected in human peripheral blood mononuclear cells (PBMC) or a well-differentiated murine macrophage cell line, J774A.1. These data indicate that PTTG1 is overexpressed in most blastic leukemia cell lines.

### PTTG1 Expression is Down-regulated during PMA-induced Myeloid Differentiation

**Figure 2:**
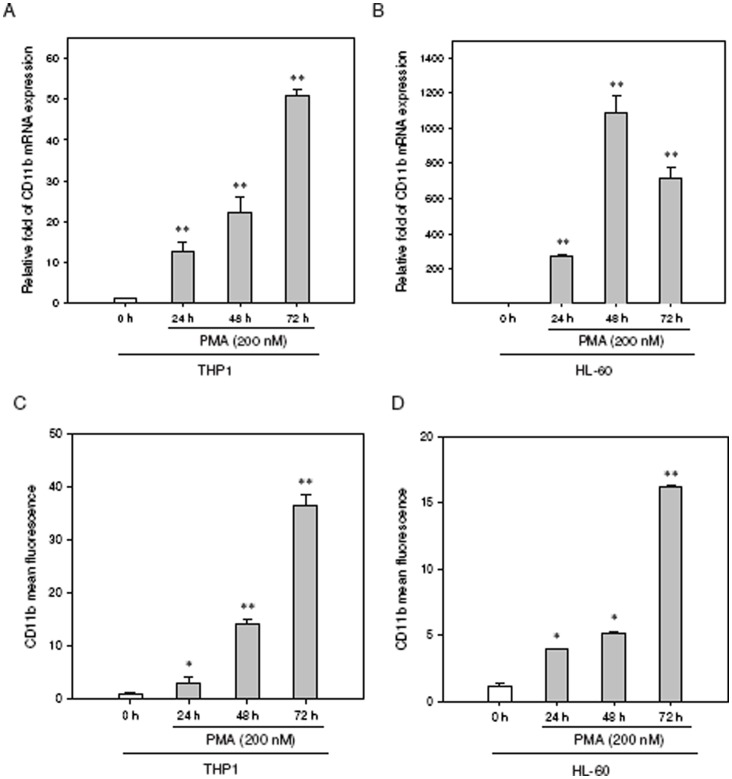
CD11b expression is induced in human leukemia cell lines upon treatment with PMA. The THP1 or HL-60 cells were treated with PMA (200 nM) for the indicated periods. The CD11b mRNA expression was determined by qRT-PCR in (A) THP1 and (B) HL-60 cells. The CD11b(+) cells were detected using FITC-labeled CD11b antibody, then analyzed by flow cytometry and expressed as the mean fluorescence intensity in (C) THP1 and (D) HL-60 cells. Data represent the mean ± SD from three independent experiments. *p<0.05 and **p<0.01 represents significant differences compared with the 0 h group.

To investigate the expression of PTTG1 in differentiated leukocytes, we assessed myeloid differentiation using PMA, a well-known agent that triggers myeloid differentiation in these cells [25], [26]. As shown in **Figure S1**, PMA (200 nM) promotes cellular adhering to the dishes. Most adherent cells were round, but some showed morphological changes indicating macrophage differentiation (pseudopod-like protrusions) in THP1 and HL-60 cells. The mRNA expression of CD11b, a marker of myeloid differentiation, was significantly up-regulated in THP1 and HL-60 cells upon treatment with PMA (**Figures 2A and 2B**). When differentiation was assessed by flow cytometric analysis of the CD11b cell surface marker, CD11b was found to be expressed in a time-dependent manner and peaked at 72 h in the PMA-induced THP1 and HL-60 cells (**Figures 2C and 2D**). These data reveal that both the THP1 and HL-60 cells can be driven to differentiate into macrophages by PMA.

**Figure 3:**
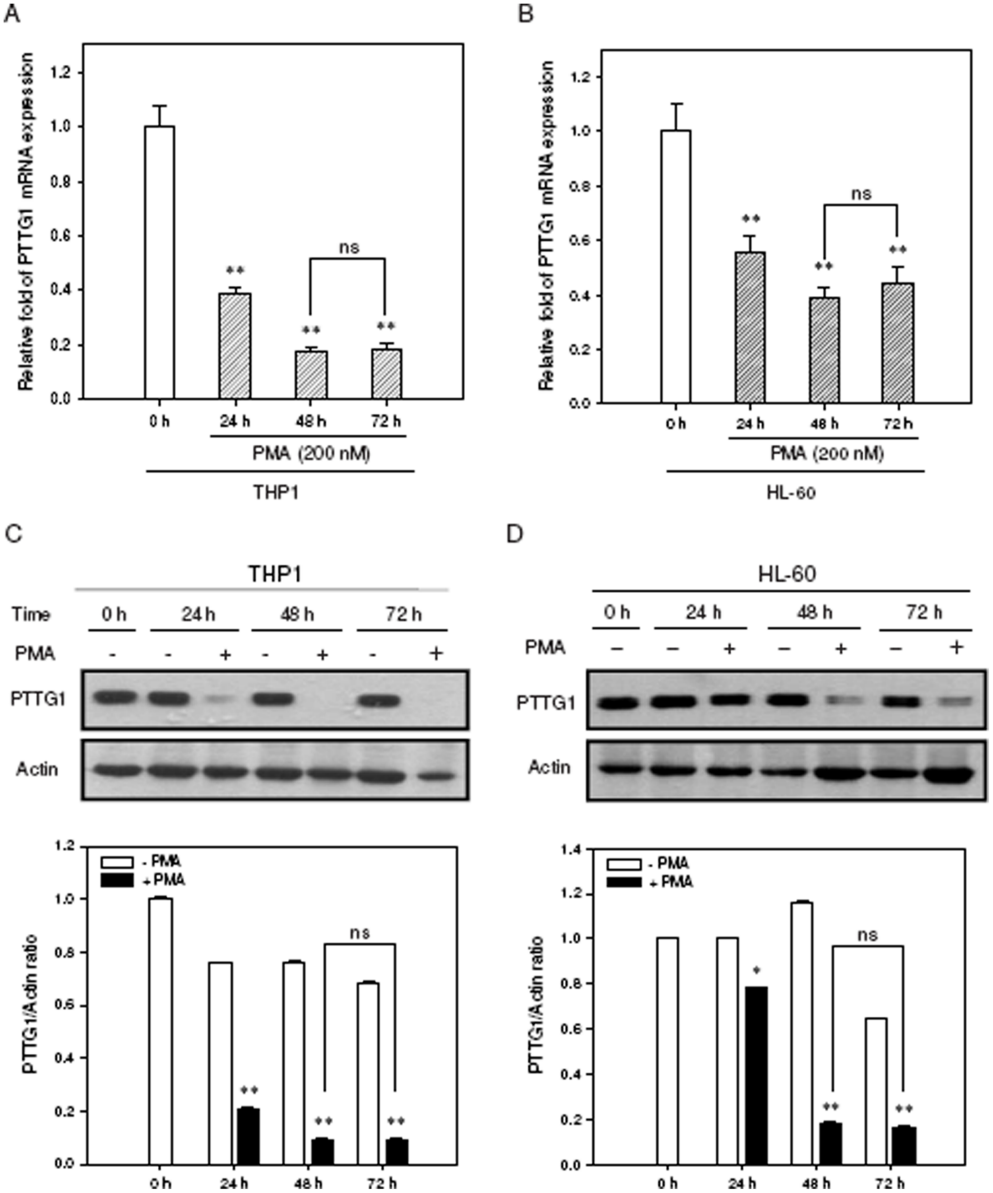
PTTG1 expression is down-regulated during PMA-induced cell differentiation. Total cellular RNA was extracted and PTTG1 mRNA expression was determined by qRT-PCR in (A) THP1 and (B) HL-60 cells treated with PMA (200 nM) for indicated periods. Data represent the mean ± SD from five independent experiments. **p<0.01 represents significant differences compared with the 0 h group. ns represents no significance. Western blot analysis was performed to detect PTTG1 and actin proteins in (C) THP1 and (D) HL-60 cells. The immunoblot experiments were replicated at least three times, and a representative blot is shown. The normalized intensity of PTTG1 versus actin is presented as the mean ± SD of three independent experiments. *p<0.05 and **p<0.01 represents significant differences compared with the 0 h group. ns represents no significance.

We then investigated the effect of PMA-induced myeloid cell differentiation on PTTG1 expression in these leukemia cell lines. THP1 and HL-60 cells were treated with PMA (200 nM) for the indicated period, and PTTG1 expression was determined by quantitative reverse transcription PCR (qRT-PCR) and Western blot analysis. As shown in **Figures 3A and 3B**, the mRNA levels of PTTG1 were significantly decreased in PMA-primed THP1 and HL-60 cells (approximately 82% and 51% reduction at 72 h, respectively). Western blot results showed the PTTG1 protein was highly expressed in PMA-untreated cells (from 0 to72 h), however, the protein levels of PTTG1 in PMA-primed THP1 and HL-60 cells were markedly reduced when compared with those of undifferentiated cells (**Figures 3C and 3D**). While these cells were treated with cycloheximide (CHX) (50 µg/ml) for indicated periods (0–24 h), the protein lysates were harvested and the half-life of PTTG1 protein was detected by Western blot analysis. The half-life of PTTG1 was near 4 h in THP1 and 3 h in HL-60 cells. In addition, the PTTG1 protein can be detected in cells treated with CHX for 24 h (**Figure S2)**. This data indicate that PTTG1 is a relatively stable protein overexpressed in undifferentiated leukemia cell lines. Furthermore, to confirm PTTG1 suppression is correlated with myeloid differentiation, the THP1 and HL-60 cells were treated with another myeloid differentiation agent, retinoic acid (RA), and gene expression was analyzed by qRT-PCR and Western blot analysis. When the cells were treated with RA (1 µM for THP1 and 5 µM for HL-60) for 48 and 72 h, the PTTG1 mRNA and protein expression were significantly reduced when compared with those of undifferentiated cells (**Figure S3**). These results showed that PTTG1 expression is down-regulated during PMA or RA induced myeloid cell differentiation.

### PTTG1 Promoter Activity is Suppressed in PMA-induced THP1 and HL-60 Toward Macrophages

**Figure 4:**
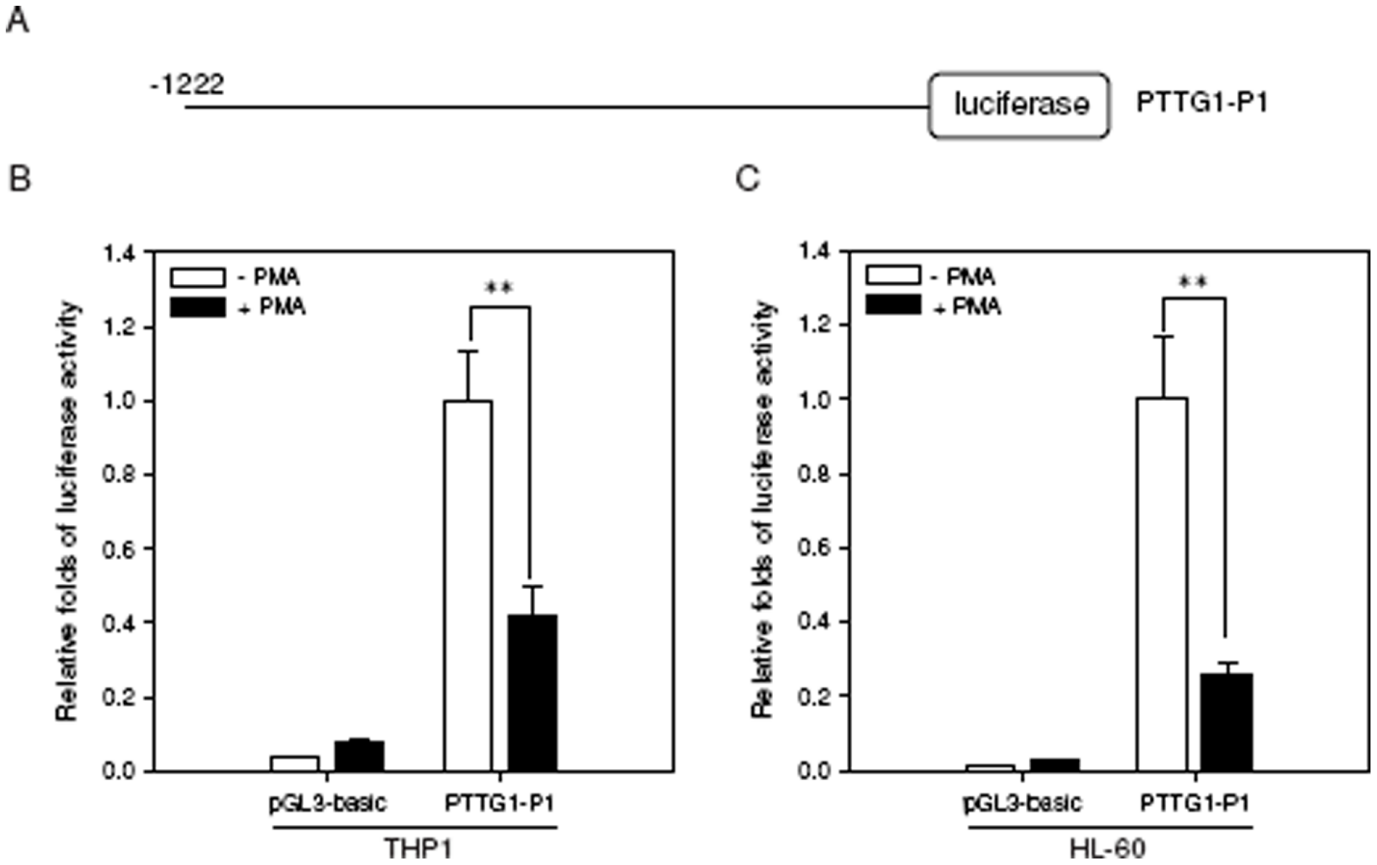
PTTG1 promoter activity is suppressed during PMA-induced THP1 or HL-60 cell differentiation. (A) Schematic diagram of a construct of PTTG1 promoter-luciferase reporter. Position +1 was assigned to the nucleotide at the transcription start site. The control pGL3-basic vector or the PTTG1-P1 construct was co-transfected with the *Renilla* internal control vector into THP1 or HL-60 cells. Twenty-four hours after transfection, cells were treated with vehicle (0.1% DMSO) or PMA (200 nM) for 24 h. Cells were then harvested for the luciferase reporter assay. PTTG1 promoter activity was detected in PMA-primed THP1 (B) and HL-60 (C) cells. The intensities of the luciferase reactions measured in the lysates of the transfected cells were normalized to their *Renilla* luciferase control activity. Data represent the mean ± SD from three independent experiments. ** *p*<0.01 represents significant differences compared with the vehicle-treated cells.

To test whether the reduction of PTTG1 expression induced by differentiation is regulated at the transcriptional level, a pGL3-basic vector lacking a promoter or a luciferase reporter construct that contains the 5′-flanking region of the PTTG1 promoter (**Figure 4A**, from −1222 to +37, relative to the transcription start point) were co-transfected with the *Renilla* internal control vector into THP1 or HL-60 cells. When THP1 and HL-60 cells were treated with PMA for 24 h, the luciferase activities were significantly decreased by approximately 60% and 75%, respectively, compared with those of cells treated with vehicle (*p*<0.01) (**Figures 4B and 4C**). This result suggests that PTTG1 promoter activity was suppressed during PMA-induced myeloid differentiation.

**Figure 5:**
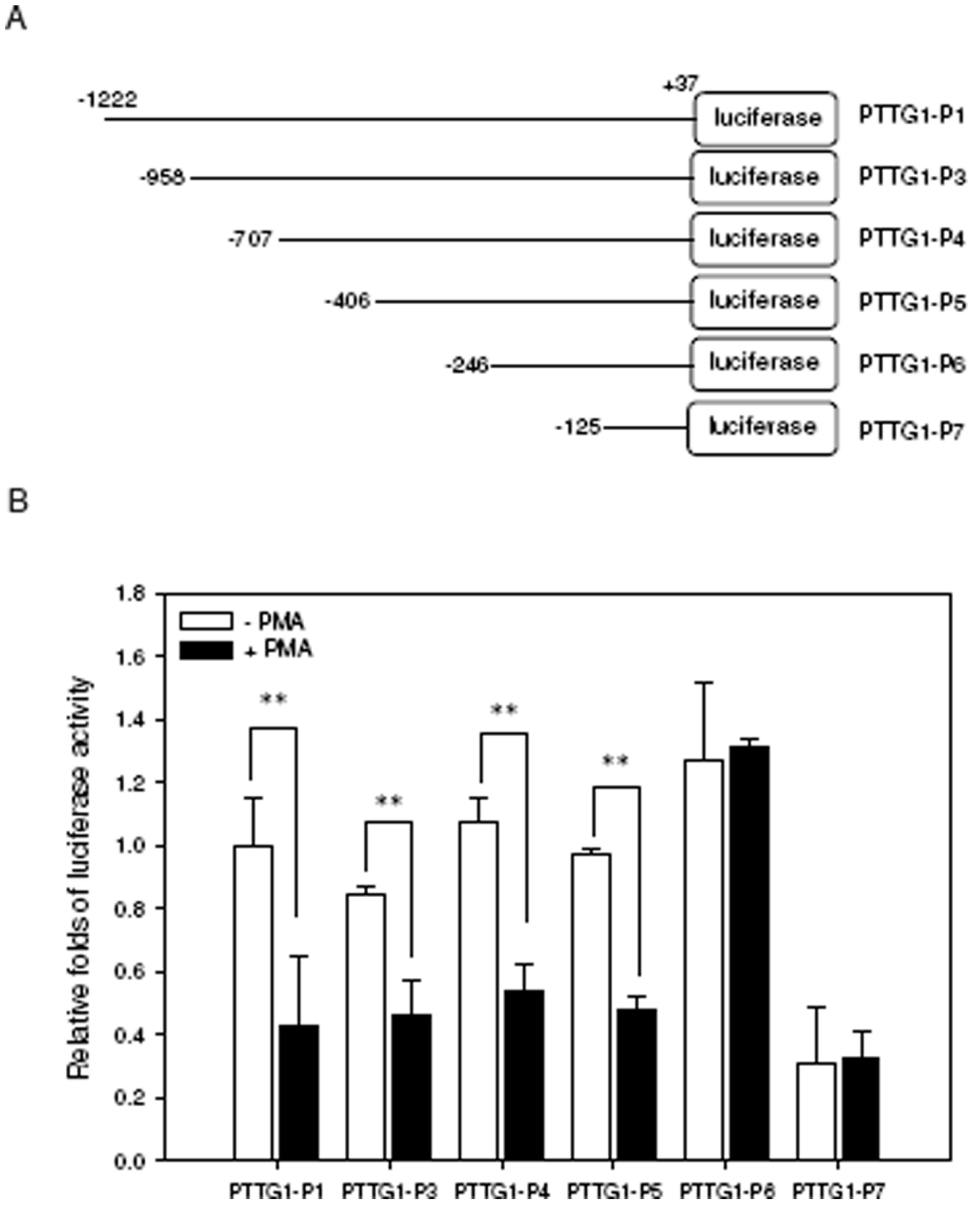
Characterization of a PMA-responsive regulatory element within the PTTG1 promoter. (A) Schematic diagram of the serial deleted constructs of PTTG1 promoter luciferase reporters. (B) The reporter constructs were transiently co-transfected with the *Renilla* luciferase control vector into THP1 cells. Twenty-four hours after transfection, cells were treated with vehicle (0.1% DMSO) or PMA (200 nM) for 24 h. Cells were then harvested for the luciferase reporter assay. The intensities of the luciferase reactions measured in the lysates of the transfected cells were normalized to their *Renilla* luciferase control activity. Data represent the mean ± SD from three independent experiments. ** *p*<0.01 represents significant differences compared with the vehicle-treated cells.

To identify the regulatory elements of the PTTG1 promoter that are responsive to PMA-induced cell differentiation, a serial deletion of PTTG1 promoter constructs (**Figure 5A**) together with *Renilla* control vector for normalization were co-transfected into THP1 cells followed by treatment with PMA for 24 h. As shown in **Figure 5B**, the luciferase activities of the PTTG1-P1 (−1222/+37), PTTG1-P3 (−958/+37), PTTG1-P4 (−707/+37) and PTTG1-P5 (−406/+37) constructs in transfected cells were significantly reduced by PMA exposure. In contrast, the promoter activities were not affected in cells transfected with the PTTG1-P6 (−246/+37) and PTTG1-P7 (−125/+37) constructs. These results indicate that the PMA-responsive elements within the PTTG1 promoter reside between the −406 and −246 positions. Sequence analysis and a previous report indicated that this region contains the transcriptional repressor Krueppel-like factor 6 (KLF6) binding element (GGCGGGG) located in the nucleotides −346 to −340 of the PTTG1 promoter [27].

### The Effect of KLF6 on the Suppression of PTTG1 Promoter Activity

**Figure 6:**
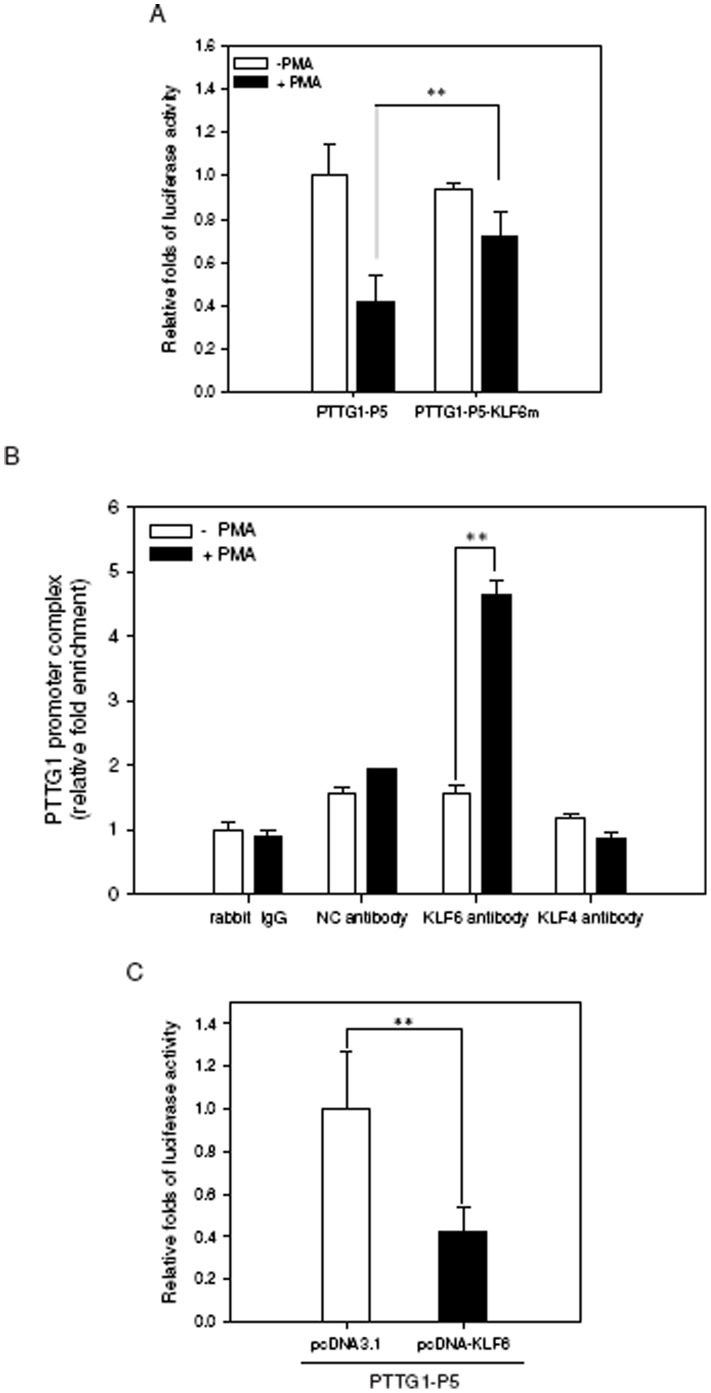
KLF6 binds to the PTTG1 promoter and represses PTTG1 transcription. (A) A mutation construct of the PTTG1 promoter (PTTG1-P5-KLF6m) contained the site-specific mutated nucleotides within the KLF6 binding element of PTTG1-P5 construct. The wild-type or mutated reporter constructs were transiently co-transfected with *Renilla* luciferase control vector into THP1 cells for 24 h. Cells were harvested and the luciferase activity was measured. The intensities of the luciferase reactions measured in the lysates of the transfected cells were normalized to the *Renilla* luciferase control activity. Data represent the mean ± SD from three independent experiments. ** *p*<0.01 represents significant differences compared with the PTTG1-P5 transfected cells followed by PMA treatment. (B) THP1 cells were treated with vehicle (0.1% DMSO) or PMA (200 nM) for 48 h followed by a ChIP assay as described in Material and Methods. After immunoprecipitation of cellular chromatin with control rabbit IgG, anti-CREB (as a KLF-unrelated antibody negative control; NC antibody), anti-KLF6 or anti-KLF4 antibody, the PTTG1 promoter complex was measured by quantitative real time PCR using primers span the nucleotides −406 to −246 region of PTTG1 promoter. Data are expressed as the fold increase of NC antibody, anti-KLF6 or anti-KLF4 antibody over the control rabbit IgG, respectively. Data represent the mean ± SD from five independent experiments. ** *p*<0.01 represents significant differences compared with the anti-KLF6 antibody without PMA treatment. (C) The pcDNA3.1(+) control vector or pcDNA-KLF6 expression plasmid were co-transfected with PTTG1-P5 reporter plasmid and *Renilla* luciferase control vector into THP1 cells. Cells were harvested 48 h post-transfection for the luciferase reporter assay. The intensities of the luciferase reactions measured in the lysates of the transfected cells were normalized to the *Renilla* luciferase control activity. Data represent the mean ± SD from three independent experiments. ** *p*<0.01 represents significant differences compared with the pcDNA3.1(+) control vector-transfected cells.

To test whether the KLF6 site is an essential regulatory element for PTTG1 transcription during PMA-induced differentiation, the PTTG1-P5-KLF6m plasmid, which contains the mutation of core KLF6 binding element nucleotides, was transfected into THP1 cells and was followed by PMA treatment. As shown in **Figure 6A**, the luciferase reporter activity during PMA-induced differentiation was not affected in cells transfected with the KLF6 mutant construct compared with THP1 cells transfected with the wild-type construct. This result implies that KLF6 may play a critical regulatory function for PTTG1 transcription in PMA-induced differentiation. To determine whether KLF6 directly interacts with the PTTG1 promoter *in vivo*, we performed a ChIP assay in PMA-treated THP1 cells. As shown in **Figure 6B**, KLF6 strongly interacts with the KLF6 binding element in PMA-treated cells, and the level of KLF6 bound to the PTTG1 promoter site was approximately 4.6-fold higher than that of cells without PMA treatment (*p*<0.01). However, PTTG1 promoter complex related to unrelated negative control antibody (NC antibody) or KLF4 antibody was not enriched in PMA-treated cells (**Figure 6B**). These results indicate that KLF6 specifically interacts with PTTG1 promoter in PMA-treated THP1 cells. In addition, we determined the functional role of KLF6 in PTTG1 transcription by transfecting the KLF6 expression construct (pcDNA-KLF6) or the mock vector (pcDNA3.1) into THP1 cells and measured the reporter activities at 48 h post-transfection. **Figure 6C** shows that the PTTG1 promoter activity was decreased by approximately 60% in pcDNA-KLF6-transfected cells compared with pcDNA3.1 vector control-transfected cells (*p*<0.01). These data demonstrate that, to some extent, KLF6 interacts with PTTG1, even without PMA treatment. Thus, KLF6 is a predominant regulatory factor for PTTG1 transcription in PMA-induced cell differentiation.

### KLF6 Expression is Up-regulated during PMA-induced Myeloid Differentiation

**Figure 7:**
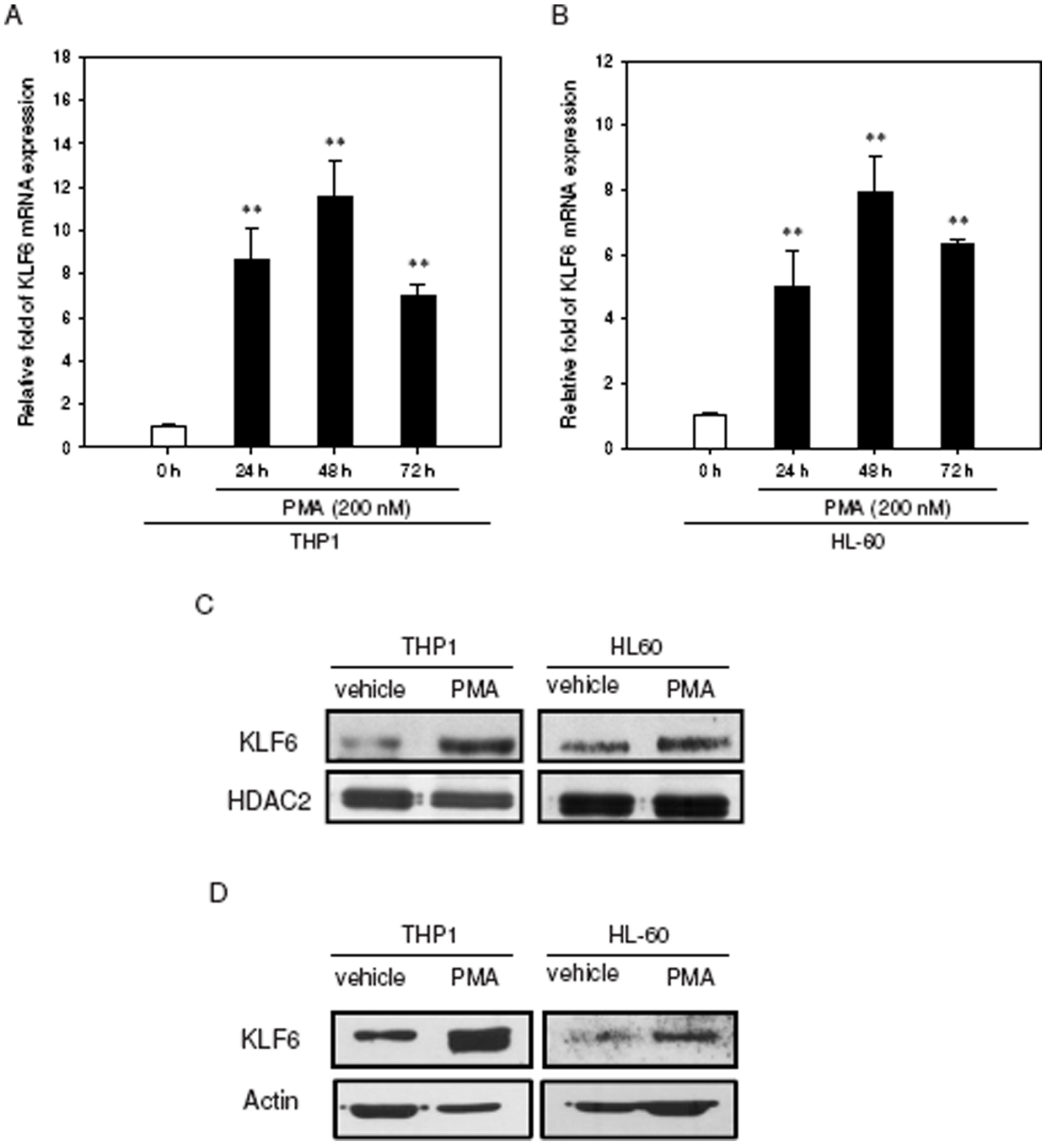
Examination of KLF6 expression levels during PMA-induced differentiation in THP1 and HL-60 cells. THP1 or HL-60 cells (1×10^6^/well) were treated with PMA (200 nM) for indicated periods. Total cellular RNA was extracted and KLF6 mRNA expression was determined by qRT-PCR in (A) THP1 and (B) HL-60 cells. Data represent the mean ± SD from five independent experiments. **p<0.01 represents significant differences compared with the 0 h group. Cells were treated with PMA (200 nM) for 48 h, and nuclear extracts (C) or cytoplasmic fractions (D) were prepared for the analysis of KLF6 protein levels. Western blot analysis was performed with antibodies against KLF6, HDAC2 and actin in THP1 and HL-60 cells. The immunoblot experiments were replicated at least three times, and a representative blot is shown.

To explore the responsiveness of the endogenous KLF6 to PMA addition, leukemia cells were treated with PMA (200 nM), and the KLF6 mRNA expression levels were determined by qRT-PCR for the indicated periods. As shown in **Figures 7A and 7B**, the levels of KLF6 transcripts increased significantly in the PMA-primed THP1 and HL-60 cells (approximately 12- and 7-fold inductions at 48 h, respectively). Increased expression of KLF6 was also observed with RA-treated THP1 and HL-60 cells (**Figure S4**).

Since PMA may induce expression of other KLFs such as KLF2 and KLF4, we next examine whether KLF2 or KLF4 expression can be up-regulated like KLF6, in both PMA-primed THP1 and HL-60 cells. As shown in **Figure S5**, when cells were treated with PMA for 24–72 h, the mRNA expression of KLF2 was down-regulated in THP1, but up-regulated in HL-60 cells in a time-dependent manner. The level of KLF4 mRNA expression increased at 24 h and quickly declined in THP1 and only slightly enhanced in HL-60 cells. This data indicate that KLF2 or KLF4 expression is not persistently up-regulated in PMA-treated THP1 and HL-60 cells. These above results reveal that KLF6 expression, not other KLFs such as KLF2 or KLF4, is significantly up-regulated in both THP1 and HL-60 cells during myeloid differentiation.

Previous studies have indicated that cell stimulation mediated by phorbol esters led to an evident increase in nuclear staining of KLF6 protein [28]. We therefore analyzed KLF6 nuclear expression by performing Western blot analysis on nuclear protein extracts. Our findings showed that the levels of KLF6 protein in nuclear extracts from the PMA-treated THP1- and HL-60-cells were significantly increased compared with those in vehicle-treated cells (**Figure 7C**). We also examined the cytoplasmic extracts and found that KLF6 protein showed increased expression in the cytoplasmic fraction (**Figure 7D**). These observations suggest that upon PMA stimulation, KLF6 mRNA is up-regulated and KLF6 protein levels are increased in both the nuclear and cytoplasmic compartments.

### Knockdown of KLF6 Expression Abolished Repression of PTTG1 by PMA

**Figure 8:**
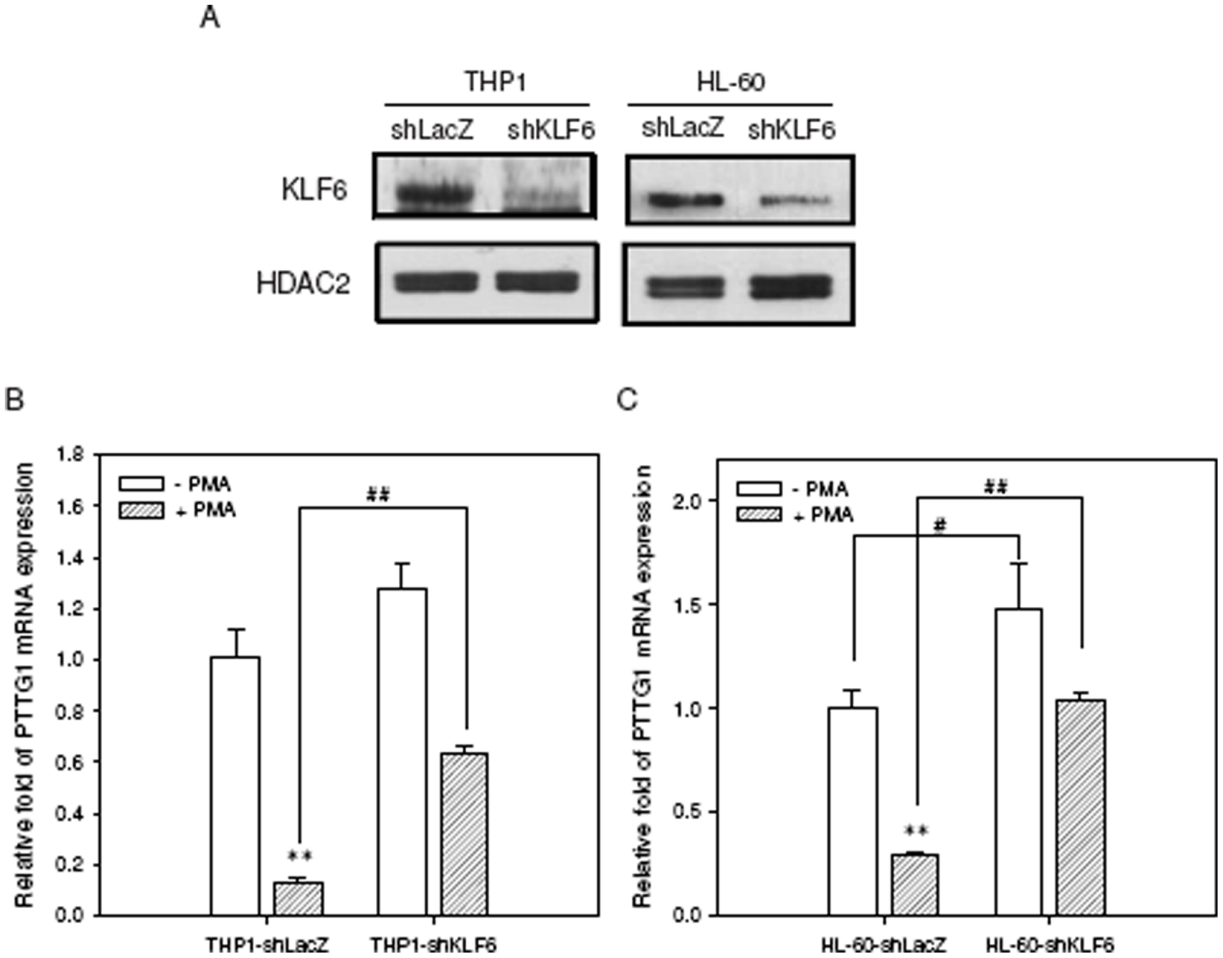
The effect of KLF6 knockdown on PTTG1 transcription during PMA-induced cell differentiation. (A) Knockdown of KLF6 expression was performed using a specific lentiviral vector encoding KLF6 shRNA, and stable clones were selected with puromycin (2 µg/ml) for 2 weeks. Western blot of nuclear extracts was performed to examine the knockdown efficiency of shKLF6-THP1 and shKLF6-HL-60 cells compared with cells transfected with shLacZ lentiviral vector. Cells with stable KLF6 knockdown were treated with PMA (200 nM) for 72 h. Total cellular RNA was extracted and PTTG1 mRNA expression was determined by qRT-PCR in (B) THP1 and (C) HL-60 cells. Data represent the mean ± SD from three independent experiments. **p<0.01 represents significant differences compared with PMA non-treated cells. ^##^ *p*<0.01 represents significant differences compared with the PMA-treated shLacZ knockdown control group.

Next, we determined the effect of shRNA-mediated KLF6 knockdown on the PTTG1and CD11b expression in PMA-primed leukemia cells. KLF6 shRNA significantly reduced the KLF6 expression in THP1 and HL-60 cells (**Figure 8A**). We first evaluated PTTG1 expression without PMA treatment. As shown in **Figures 8B and 8C**, shRNA-mediated KLF6 silencing alone enhanced PTTG1 expression in comparison with shLacZ control cells. Upon treatment with PMA, the shKLF6 cells remarkably increased the expression of PTTG1 relative to shLacZ cells (*p*<0.01). Moreover, the induction of differentiation marker CD11b in PMA-primed cells was significantly reduced after KLF6 knockdown (**Figure S6**). These results indicate that KLF6 plays an important role in the down-regulation of PTTG1 expression during PMA-induced THP1 and HL-60 cell differentiation.

### PKC and MAPK/ERK Pathways are Involved in the Down-regulation of PTTG1 After PMA Treatment

**Figure 9:**
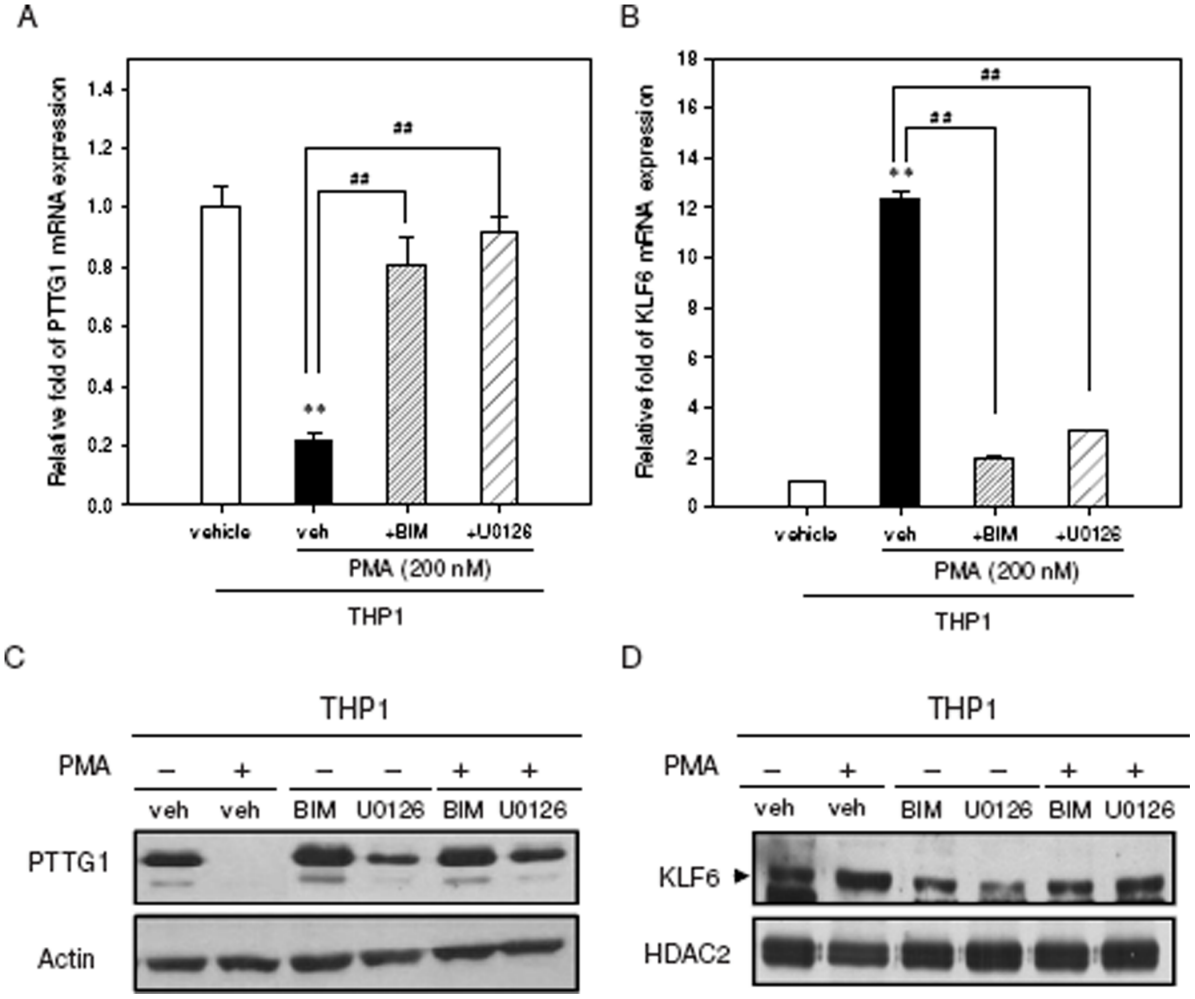
Signaling pathways involved in the down-regulation of PTTG1 during PMA-induced THP1 cell differentiation. THP1 cells were pretreated with vehicle (veh, 0.1% DMSO), bisindolylmaleimide I (BIM, 5 µM) or U0126 (10 µM) for 30 min followed by exposure to PMA (200 nM) for 48 hr. (A) Total cellular RNA was extracted and PTTG1 mRNA expression was determined by qRT-PCR. (B) Total cellular RNA was extracted and KLF6 mRNA expression was determined by qRT-PCR. Data represent the mean ± SD from three independent experiments. ** *p*<0.01 represents significant differences compared with PMA-non-treated cells. ^##^ *p*<0.01 represents significant differences compared with the vehicle group. (C) The PTTG1 protein from total cellular lysate was detected by Western blot analysis. (D) The level of KLF6 protein from nuclear extract was determined by Western blot analysis. The immunoblot experiments were replicated at least three times, and a representative blot is shown.

PMA is known to activate protein kinase C (PKC) and MAPK/ERK pathways during the induction of cell differentiation in PMA-treated THP1 and HL-60 cells [29], [30], [31]. Consequently, we investigated whether PTTG1 down-regulation was dependent on the PKC and MAPK/ERK pathways in PMA-primed leukemia cells. THP1 and HL-60 cells were treated with the PKC inhibitor bisindolylmaleimide I (BIM) or the MAPK/ERK kinase 1/2 (MEK1/2) inhibitor U0126 for 30 min before addition of PMA for 48 h, and the PTTG1 and KLF6 expression levels were then analyzed. As shown in **Figure 9**, when THP1 cells were treated with PMA in combination with BIM or U0126, the PMA-mediated suppression of PTTG1 and induction of KLF6 were both potently inhibited. The PTTG1 mRNA and protein levels in PMA-primed leukemia cells were increased when BIM and U0126 were used (**Figures 9A and 9C**). We also observed a concomitant reduction of KLF6 mRNA and protein (**Figures 9B and 9D**). Treatment with BIM or U0126 alone did not affect PTTG1 or KLF6 expression (**Figures 9C and 9D**). Very similar effects of BIM and U0126 were also observed in HL-60 cells (**Figure S7**). However, the treatment of cells with JNK inhibitor SP600125 or p38/MAPK inhibitor SB203580 did not alter the levels of PTTG1mRNA and protein in PMA-induced differentiation (data not shown). These data indicate that PKC and MAPK/ERK pathways play essential roles in the regulation of PTTG1 expression during PMA-induced monocyte/macrophage differentiation.

## Discussion

**Figure 10:**
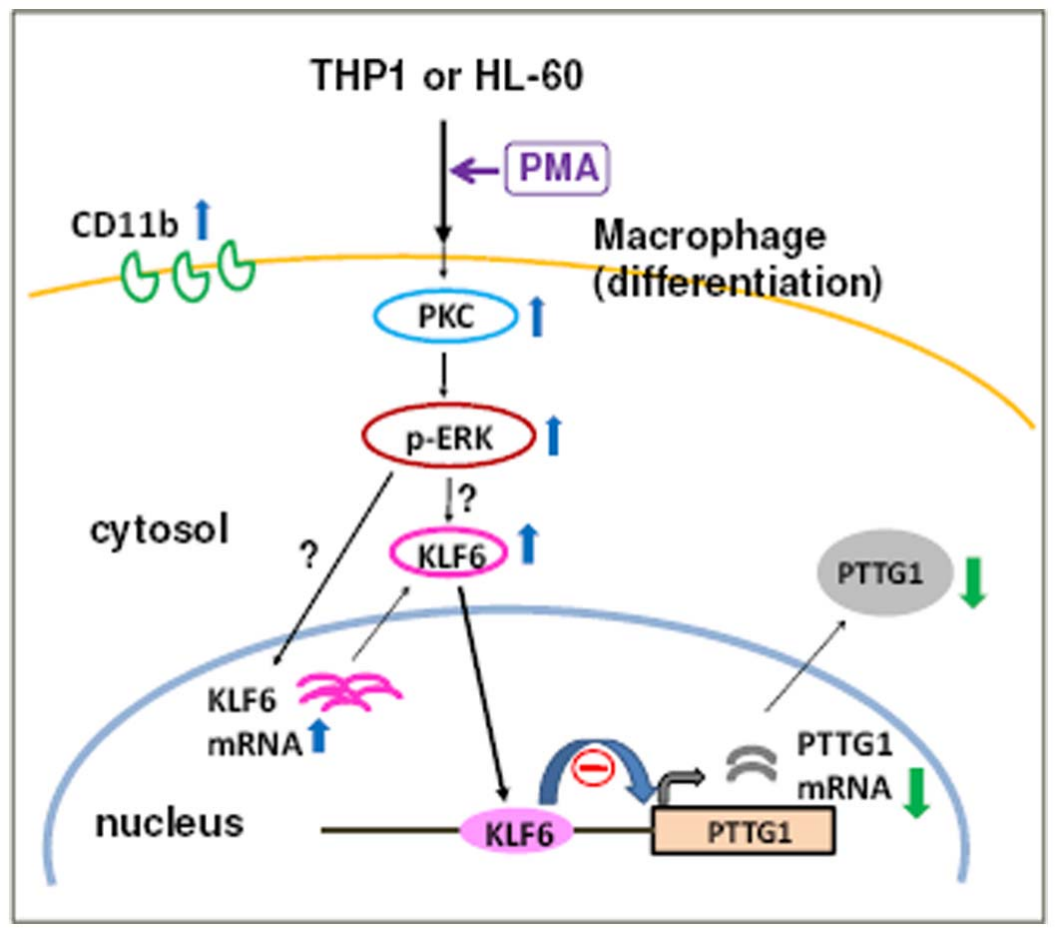
Proposed model for the down-regulation of PTTG1 during PMA-induced leukemia cell differentiation. In THP1 and HL-60 cells, the activation of PKC/ERK/KLF6 signaling axes contributes to PTTG1 suppression during PMA-induced myeloid differentiation.

AML is characterized by an increasing amount of morphologically immature and undifferentiated blood cells that interfere with normal hematopoiesis due to their active proliferation. Exploring the signaling molecules involved in myeloid differentiation is important both for understanding hematopoietic development and for developing therapeutics for AML. In this study, we demonstrated that the oncogene PTTG1, which is overexpressed in leukemia cells, is transcriptionally repressed by the tumor suppressor KLF6 in response to PMA-induced myeloid differentiation. Moreover, the activation of PKC and MAPK/ERK by PMA is important for the increase in KLF6 expression that, in turn, down-regulates PTTG1 transcription (**Figure 10**).

PTTG1 overexpression has been reported to correlate with tumor development in various types of hematopoietic neoplasms [5], [21]. In this study, we showed that PTTG1 is abundantly expressed in several leukemia cell lines; in contrast, its expression in normal PBMCs and well-differentiated macrophages is undetectable. Our findings are in agreement with a previous report describing the differential expression of PTTG1 proteins in hematopoietic malignancies [5]. The detailed molecular mechanism involved in PTTG1 overexpression in these leukemia cells remains unclear. In acute leukemias, genes coding for transcription factors are the most frequently affected by altered expression [32]. Transcriptional activators such as Sp1, nuclear factor-Y and β-catenin/T cell factor (TCF) have been implicated in the activation of human PTTG1 expression via the regulation of its promoter activity in various cancer cells [33], [34], [35], [36]. In addition, several growth factors, such as estrogen, insulin, IGF-I, EGF, TGFα and HGF, have been reported to contribute to PTTG1 induction [7], [37], [38], [39]. However, very few transcriptional repressors for PTTG1 expression and their related signaling pathways have been reported. In this study, we clearly demonstrated that PTTG1 gene expression is significantly down-regulated through the inhibition of PTTG1 promoter activity in PMA-primed THP1 or HL-60 leukemia cells. Furthermore, the PMA-responsive region that we identified is located at nucleotides −406 to −246 upstream of the PTTG1 promoter and contains a binding site for KLF6, which is a zinc-finger transcription factor with tumor suppressor function [27], [40], [41], [42].

KLF6 is a transcription factor belonging to the Sp/KLF superfamily and may preferentially binds to GC-rich regions on diverse target genes. Several GC-rich regions in the PTTG1 promoter have been previously characterized in response to Sp1 and NF-Y, but also putative binding sites for KLF6 [34]. Lee *et al.* had reported that KLF6 specifically and directly interacted with PTTG1 promoter within the region −246 to −401 nt, which contained a GC-box (GGCGGGG) located in the nucleotides −346 to −340, in HepG2 cell line [27]. In this study, our reporter assay showed that the PMA-responsive elements within the PTTG1 promoter reside between the −406 and −246 positions. Furthermore, when PTTG1-P5-KLF6m plasmid containing the mutated suquence (GGCGGGG to aaaGaaG) was transfected into THP1 cells and followed by PMA treatment, the reporter activity was not affected when compared with cells transfected with the wild-type construct. Moreover, ChIP assay result demonstrated increased KLF6/PTTG1 complex formation within the nucleotides −406 to −246 of PTTG1 promoter was specific in PMA-treated THP1 cells. Taken together, the GGCGGGG site within the region −246 to −401 nt of PTTG1 promoter may serve as a KLF6 binding element for transcription repression during myeloid cell differentiation.

Recent studies have indicated that Kruppel-like factors (KLFs) may be among the key regulatory factors contributing to the orchestration of several aspects of leukocyte biology [43]. Leukocyte development requires the coordination of stage-specific transcription factors to help orchestrate the processes by which a progenitor cell emerges as a functional leukocyte. Since the role of KLF6 in primary cell differentiation is not well studied, we have analyzed the KLF6 expression in human cord blood-derived CD34 (+) hematopoietic stem cells (HSC) during PMA-induced differentiation. Our data showed that the level of KLF6 mRNA was significantly up-regulated in primary human cells differentiation (data not shown). KLF6 is frequently inactivated in human cancer and has significant roles in cellular proliferation, apoptosis and differentiation [42]. The deletion of *klf6* in mice is embryonic-lethal and shows aberrant hematopoiesis. Conversely, the forced expression of KLF6 enhanced the hematopoietic potential of wild-type embryonic blood cells [44]. Moreover, Humbert et al revealed that KLF6 expression is inhibited in primary AML samples from patients compared with mature neutrophils from healthy donors [45]. Their study showed that KLF6 expression was induced during the neutrophil differentiation of acute promyelocytic leukemia (APL) cells and that inhibiting KLF6 attenuated their differentiation. In this study, we found a specific binding element for KLF6 in the PTTG1 promoter, and KLF6 binding to the PTTG1 promoter region led to the transcriptional suppression of PTTG1. Our findings suggest that increases in KLF6 binding promote the transcriptional repression of PTTG1 during PMA-induced myeloid differentiation. Our observation is consistent with a recent report describing PTTG1 as one of the most up-regulated genes in comparative gene expression profiles between KLF6+/+ and KLF6+/− murine livers [27]. That study demonstrated the reciprocal expression of KLF6 and PTTG1 both in hepatocellular carcinoma (HCC) patient samples and in the HepG2 cell line, suggesting that the loss of KLF6 and the subsequent up-regulation of PTTG1 could contribute to tumorigenesis in HCC. Our current study showed the transcriptional repression of PTTG1 during myeloid differentiation and identified the suppressive role of KLF6 in regulating PTTG1 transcription. In addition, we showed that the activation of PKC/ERK signaling axes contributed to KLF6 induction and PTTG1 suppression.

It is well known that PMA can activate the PKC-dependent MAPK/ERK pathway, induce growth arrest and induce differentiation into monocytes/macrophages in leukemia cells such as THP1 and HL-60 [31], [46]. In this study, the PKC inhibitor bisindolylmaleimide I and the MAPK/ERK kinase 1/2 (MEK1/2) inhibitor U0126 significantly blocked the potentiation of PMA-mediated KLF6 induction and the down-regulation of PTTG1. Thus, it appears that PTTG1 suppression in PMA-primed leukemia cells goes through the activation of the PKC/ERK/KLF6 pathway. Treatment of THP1 or HL-60 cells with inhibitors of JNK or p38 prior to PMA addition did not affect the down-regulation of PTTG1 at either the mRNA or the protein levels (data not shown). However, in a non-small cell lung cancer study, PKC has been associated with KLF6 activation following PMA-mediated growth arrest. And the study also showed that the activation of JNK pathways is a downstream effector for KLF6 protein induction [47]. Therefore, the effect of PMA appeared to be dependent on either cell type or context. Thus, the regulatory effect of other signaling cascades on the KLF6 protein warrants further investigation.

The transformation of a normal cell to a tumor cell appears to depend in part on alterations in genes that control cell cycle, thereby resulting in inappropriate cellular survival and tumorigenesis. The expression of PTTG1, a mammalian securin protein, is characterized as being cell cycle-dependent [9], [48], [49]. PTTG1 also plays an important role in the control of cell cycle progression by activating cell cycle regulators such as cyclin D3 and c-myc and by repressing cell cycle inhibitor p21 [11], [50], [51]. Interestingly, KLF6 inhibits cell cycle progression through several pathways, including the transactivation of p21, the inhibition of c-Jun and the disruption of cyclin D1/CDK4 complexes, resulting in growth arrest [28], [52], [53]. Thus, it is likely that KLF6 expression is induced to trigger PTTG1 suppression and inhibit cell proliferation. Although hematopoietic differentiation is associated with a loss of proliferative capacity, the differentiation of leukemia cells may not result in the cessation of growth. Our data also showed that KLF6 affects cellular differentiation because the induction of the differentiation marker CD11b in PMA-primed cells was significantly decreased by KLF6 knockdown. In addition, Rovera et al [54] demonstrated that PMA-treated HL-60 cells became strongly positive for monocytic markers, independent of DNA synthesis and cell division. Therefore the inhibition of PTTG1 transcription by KLF6 may couple anti-proliferation with the promotion of differentiation. Additional studies will help validate the role of PTTG1 in leukemia differentiation and its effects on tumorigenesis.

In conclusion, we demonstrated that the overexpression of PTTG1 could be repressed by KLF6 tumor suppressor through the activation of PKC/ERK signaling in myeloid cell differentiation. Because PTTG1 overexpression promotes tumorigenesis and metastasis and may maintain the precursor stages of leukemia cells, PTTG1 knockdown could be a potential option for cancer therapy. Further characterization of PTTG1 suppression and its related intracellular signaling pathways may provide the basis for therapy. Our results suggest that drugs that increase the activities of KLF6 may inhibit the transcription of PTTG1 and have the potential to be used as a therapeutic tool for hematopoietic diseases.

## Materials and Methods

### Chemicals

Phorbol 12-myristate 13-acetate (PMA), retinoic acid (RA), cycloheximide (CHX), and dimethyl sulfoxide (DMSO) were purchased from Sigma-Aldrich Co. (St. Louis, MO, USA). Bisindolylmaleimide I (BIM), a protein kinase C inhibitor, was purchased from Cayman Chemical (Ann Arbor, MI, USA). U0126 [1,4-diamino-2,3-dicyano-1,4-bis (2-aminophenylthio)-butadiene], a selective inhibitor of MAPK/ERK kinase 1/2 (MEK1/2), was purchased from Promega (Madison, WI, USA).

### Cell Lines and Establishing KLF6 Knockdown Stable Clones

The following cell lines were used in this study: HL-60 (human acute promyelocytic leukemia cell line), THP1 (human acute monocytic leukemia cell line), U937 (human histiocytic lymphoma cell line), K-562 (chronic myelogenous leukemia cell line), HEL 92.1.7 (human erythroleukemia cell line), RAW264.7 (mouse monocyte/macrophage cell line) and J774A.1 (mouse reticulum cell sarcoma cell line) (from ATCC, Manassas, VA, USA). These cell lines were maintained in complete medium containing RPMI-1640 (Sigma-Aldrich), 2 mM glutamine, 1.5 g/L sodium bicarbonate, 4.5 g/L glucose, 10 mM HEPES and 1 mM sodium pyruvate and supplemented with 10% fetal bovine serum (FBS) (Biological Industries, Kibbutz Haemek, Israel) in a 5% CO_2_ incubator at 37°C. The control LacZ- and KLF6-knockdown THP1 or HL-60 cells were generated by lentiviruses encoding specific shRNA reagents obtained from the National RNAi Core Facility, Academia Sinica (Taipei, Taiwan) (LacZ-shRNA clone: TRCN0000072233; KLF6-shRNA clone: TRCN0000013711). To create the stable knockdown clones, cells were selected with puromycin (2 µg/ml) for further use and characterized by Western blot analysis.

### Western Blotting Analysis

HL-60 or THP1 cells (1×10^6^/well) were seeded on 6-well plates in RPMI containing 10% fetal bovine serum and treated with vehicle (0.1% DMSO) or PMA (200 nM) for the indicated periods. To determine the half-life of PTTG1 proteins, the cells were treated with cycloheximide (CHX) (50 µg/ml) for indicated periods. For total cellular protein extraction, the cells were washed with PBS before extraction of proteins using ice-cold cell lysis buffer (iNtRON Biotechnology, Seoul, Korea). The resulting lysates were centrifuged at 12,000×g for 10 min at 4°C, and the supernatants were carefully transferred to a microcentrifuge tube. To isolate nuclear and cytoplasmic extracts, these fractions were harvested using NE-PER nuclear and cytoplasmic extraction reagent (Thermo Fisher Scientific, Rockford, IL, USA) according to the manufacturer’s instructions. The protein concentration was measured by the Bradford method (Bio-Rad Laboratories, Hercules, CA, USA), using bovine serum albumin as the standard. Total cell lysates (60 µg), nuclear extract (20 µg), or cytoplasmic fractions (60 µg) were separated on 12% SDS-PAGE and transferred onto a PVDF membrane (PerkinElmer, Boston, MA, USA) at 15 V overnight at 4°C. The membranes were incubated at 4°C in PBST blocking buffer (5% non-fat dried milk in PBS containing 0.1% Tween-20) for 8 h. Then, the blots were incubated with the appropriate antibodies overnight at 4°C: anti- PTTG1 (1∶3,000) (Invitrogen, Carlsbad, CA, USA), anti-KLF6 (1∶3,000) (Sigma), anti-HDAC2 (1∶1,500) (GeneTex, Irvine, CA, USA), or anti-actin (1∶5,000) (Santa Cruz Biotechnology, Santa Cruz, CA) in PBST containing 1% non-fat milk overnight at 4°C. After washing with PBST, the blots were incubated with the appropriate horseradish peroxidase-conjugated secondary antibodies: anti-rabbit IgG (1∶3,000) or anti-goat IgG (1∶5,000) (Santa Cruz Biotechnology) for 1 h at room temperature. The blots were washed with PBST, and the proteins were detected by Western Lightning™ Chemiluminescence Reagent *Plus* (PerkinElmer) according to the manufacturer’s instructions. The chemiluminescence signal was visualized with X-ray film (KODAK).

### Quantitative Reverse Transcription-PCR (qRT-PCR)

Total cellular RNA was prepared using Total RNA mini Kit (Geneaid, Taipei, Taiwan). The reverse transcription of 2 µg RNA was performed using revertAid™ H minus first strand cDNA synthesis kit (Fermentas, Burlington, CA, USA). qRT-PCR was performed with 2 µL of the cDNA obtained above in 20 µL reaction mixture containing 500 nM primers [PTTG1, 5′-AAAGCTCTGTTCCTGCCTCA-3′ (forward) and 5′-GAGAGGCACTCCACTCAAGG-3′ (reverse); β-actin, 5′-TCCCTGGAGAAGAGCTACGA-3′ (forward) and 5′-AGCACTGTGTTGGCGTACAG-3′ (reverse); CD11b, 5′-ACTTGCAGTGAGAACACGTATG-3′ (forward) and 5′-AGAGCCATCAATCAAGAAGGC-3′ (reverse); KLF6, 5′-CGGACGCACACAGGAGAAAA-3′ (forward) and 5′-CGGTGTGCTTTCGGAAGTG-3′ (reverse) [27]; KLF2, 5′-CACCAAGAGTTCGCATCTGA-3′ (forward) and 5′-CGTGTGCTTTCGGTAGTGG-3′ (reverse); KLF4, 5′-CAAGTCCCGCCGCTCCATTACCAA-3′ (forward) and 5′-CCACAGCCGTCCCAGTCACAGTGG-3′ (reverse)] and Maxima SYBR Green/ROX qPCR Master Mix (Fermentas). Amplification was conducted in a Roche LightCycler®-480 Real-Time PCR System. PCR conditions were as follows: 50°C for 2 min, 94°C for 4 min, 45 cycles at 94°C for 10 sec, 58°C for 10 sec, and 72°C for 10 sec. The ΔΔC_t_ method was used for the data analysis of the indicated mRNA expression levels estimated with triplicate samples and normalized to β-actin expression levels.

### Detection of CD11b Expression by Flow Cytometry

HL-60 or THP1 cells (1×10^6^/well) were seeded on 6-well plate in RPMI containing 10% fetal bovine serum and treated with vehicle (0.1% DMSO) or PMA (200 nM) for indicated periods. Cells were washed with PBS and incubated in 300 µL PBS supplement with 5% bovine serum albumin for 60 min at 37°C. A Fluorescein isothiocyanate (FITC)-conjugated anti-human CD11b monoclonal antibody (eBioscience, San Diego, CA, USA) was added and incubated for 30 min in the dark at 4°C. After washing with PBS, the cells were suspended in 500 µL PBS, measured on the FACSCalibur and analyzed by Cell Quest Pro software (BD Biosciences, San Jose, CA, USA). CD11b expression on the cell surface was calculated by the percentage of the FITC-positive cells.

### Preparation of Reporter Constructs and KLF6 Expression Construct

The 5′ regulatory sequences of PTTG1 from nucleotide -1222 to +37, which spans from 1222 base-pairs (bp) upstream to 37 bp downstream of the transcription start site, was PCR-amplified using the human genomic DNA as a template and inserted into the pGL3-basic vector (Promega). This reporter construct of the PTTG1 promoter was designated as pPTTG-P1. The 5′ deletion constructs (PTTG1-P3, -P4, -P5, -P6, -P7) were generated using the specific primers and PTTG1-P1 plasmid as a template in PCR. These DNA fragments were amplified using the following oligonucleotides, PTTG1-P1 forward 5′-GGTACCAACAGGAAAAGGTCGTCAAC-3′; PTTG1-P3 forward 5′-GGTACCTAGAAACAGTGTCTTAGCC-3′; PTTG1-P4 forward 5′-GGTACCTGTTATTGAGTCAGTACCTC-3′; PTTG1-P5 forward 5′-GGTACCGGCTGTTAAGACCTGCGTGA-3′; PTTG1-P6 forward 5′-GGTACCTGAGCGTGGTCTCGGACTGC-3′; PTTG1-P7 forward 5′-GGTACCATTAAGTACTTGTTGGCTCA-3′; and reverse primer PTTG1-Pro-R2 5′-AAGCTTCTGGATTATTCTAAGAATG-3′. The fragment was transferred to pGL3 basic vector between KpnI and Hind III sites. The KLF6 site mutant construct (PTTG1-P5-KLF6 m) was generated by PCR using PTTG1-P5 as template and the following oligonucleotide: 5′-GTTGAGCCGGCTCCaaaGaaGAAGGAGGCG-3′ (forward) (the small letters are mutated nucleotides) as the mutant primer, and then the PCR DNA fragment was inserted into the pGL-3 basic vector. To generate the pcDNA-KLF6 expression plasmid, KLF6 was amplified by RT-PCR using the cDNA of THP1 cells and subcloned into the pcDNA3.1 vector (Invitrogen). All the above constructs were verified by DNA sequencing.

### Transfection and Luciferase Reporter Assay

HL-60 or THP1 cells (8×10^5^/well) were seeded on 6-well plates and co-transfected with the indicated PTTG1 promoter reporter constructs and *Renilla* luciferase vector (Promega) using TransIT® -2020 Transfection Reagent (Mirus Bio, Madison, WI, USA) according to the manufacturer’s instructions. Twenty-four hours after transfection, cells were treated with 200 nM PMA for 24 h and harvested using Passive Lysis Buffer (Promega). For KLF6 expression construct, THP1 cells were co-transfected with the pcDNA3.1 or pcDNA-KLF6 expression plasmid, PTTG1-P5 and *Renilla* luciferase vector using TransIT® -2020 Transfection Reagent. After 48 h culture, cells were harvested using Passive Lysis Buffer. Luciferase activities were determined using the Dual-Luciferase Reporter Assay System Kit (Promega) according to the manufacturer’s instructions. The intensities of the luciferase reactions measured in the lysates of the transfected cells were normalized to the activity of *Renilla* luciferase, which was used as an internal control.

### Chromatin Immunoprecipitation (ChIP) Assay

THP1 cells (5×10^5^/ml) were seeded in 10 cm dishes in RPMI containing 10% fetal bovine serum and treated with vehicle (0.1% DMSO) or PMA (200 nM) for 48 h. The ChIP assay was performed using the ChIP Assay Kit (Millipore, Billerica, MA, USA) in accordance with the manufacturer’s instructions. The chromatin preparation and immunoprecipitation reaction was carried out as previously described [18]. Briefly, the chromatin preparation was sheared by sonication and 1% of the sheared products were collected as an input control. The remaining chromatin preparation was incubated with anti-KLF6 (1∶100) (Sigma), anti-KLF4 (1∶100) (GeneTex), anti-CREB (1∶100; as an unrelated antibody negative control) or rabbit IgG (1∶500) (Cell Signaling Technology, Danvers, MA, USA) for 24 h at 4°C. Real-time PCR was performed to enrich promoter binding levels, and these data were expressed as fold enrichment (fold increase over the control IgG). PCR was carried out with the following KLF6 ChIP primers: 5′-CGGCTGTTAAGACCTGCGTGA-3′ (forward) and 5′-CTTAGATGGCTCCGAGCCCGTT-3′ (reverse).

### Statistical Analysis

All experiments were repeated at least three times. All values are expressed as the mean ± SD. The results were analyzed using Student’s unpaired *t*-test, and a *p* value <0.05 was considered significant.

## Supporting Information

Figure S1 **PMA induces cell differentiation in THP1 and HL-60 cells.** THP1 or HL-60 cells were seeded on 6-well plates in RPMI containing 10% fetal bovine serum and treated with PMA (200 nM) for 24, 48 and 72 h. Cell morphology was observed under a phase-contrast microscope and photographed by a digital camera. Scale bar, 200 µm.
(TIF)

Figure S2 **Measurement of the half-life of PTTG1 proteins in THP1 and HL-60 cells.** THP1 or HL-60 cells (1×10^6^/well) were seeded on 6-well plates in RPMI containing 10% fetal bovine serum and treated with cycloheximide (CHX) (50 µg/ml) for indicated periods. The PTTG1 and actin proteins from total cellular lysate were detected by Western blot analysis in THP1 (A) and HL-60 (B). The immunoblot experiments were replicated at least three times, and a representative blot is shown. Relative fold of PTTG1 (normalized intensity of PTTG1 versus actin) is presented as the mean ± SD from three independent experiments and compared with 0 h group.
(TIF)

Figure S3 **PTTG1 expression is down-regulated during retinoic acid (RA)-induced cell differentiation.** Total cellular RNA was extracted and PTTG1 mRNA expression was determined by qRT-PCR in (A) THP1 and (B) HL-60 cells treated with retinoic acid (RA; 1 µM for THP1 and 5 µM for HL60) for indicated periods. Data represent the mean ± SD from five independent experiments. *p<0.05 and **p<0.01 represents significant differences compared with the 0 h group. (C) Western blot analysis was performed to detect PTTG1 and actin proteins in THP1 and HL-60 cells. The immunoblot experiments were replicated at least three times, and a representative blot is shown.
(TIF)

Figure S4 **KLF6 expression is up-regulated during retinoic acid (RA)-induced cell differentiation.** Total cellular RNA was extracted and KLF6 mRNA expression was determined by qRT-PCR in (A) THP1 and (B) HL-60 cells treated with retinoic acid (RA; 1 µM for THP1 and 5 µM for HL60) for indicated periods. Data represent the mean ± SD from five independent experiments. **p<0.01 represents significant differences compared with the 0 h group.
(TIF)

Figure S5 **Examination of KLF2 and KLF4 mRNA expression levels during PMA-induced differentiation in THP1 and HL-60 cells.** THP1 or HL-60 cells (1×10^6^/well) were treated with PMA (200 nM) for indicated periods. Total cellular RNA was extracted and KLF2 or KLF4 mRNA expression was determined by qRT-PCR in (A) THP1 and (B) HL-60 cells. Data represent the mean ± SD from five independent experiments. *p<0.05 and **p<0.01 represents significant differences compared with the 0 h group.
(TIF)

Figure S6 **The effect of KLF6 knockdown on the CD11b mRNA expression during PMA-induced cell differentiation.** Stable shLacZ or shKLF6 knockdown clonal lines were treated with PMA (200 nM) for 72 h. Total cellular RNA was extracted and CD11b mRNA expression was determined by qRT-PCR in (A) THP1 knockdown cells and (B) HL-60 knockdown cells. Data represent the mean ± SD from three independent experiments. ^##^ *p*<0.01 represents significant differences compared with the PMA-treated shLacZ knockdown control group.
(TIF)

Figure S7 **PKC and ERK signaling pathways are involved in the down-regulation of PTTG1 during PMA-induced HL-60 cell differentiation.** HL-60 cells were pretreated with vehicle (veh, 0.1% DMSO), bisindolylmaleimide I (BIM, 5 µM) or U0126 (10 µM) for 30 min followed by exposure to PMA (200 nM) for 48 h. (A) Total cellular RNA was extracted and PTTG1 mRNA expression was determined by qRT-PCR. (B) Total cellular RNA was extracted and KLF6 mRNA expression was determined by qRT-PCR. Data represent the mean ± SD from three independent experiments. ** *p*<0.01 represents significant differences compared with PMA-non-treated cells. ^##^ *p*<0.01 represents significant differences compared with the vehicle group. (C) The PTTG1 protein levels from total cellular lysates were detected by Western blot analysis. (D) The levels of KLF6 protein from nuclear extracts were determined by Western blot analysis. The immunoblot experiments were replicated at least three times, and a representative blot is shown.
(TIF)
