# Supplementary figures and images for: Down-Regulation of the Oncogene PTTG1 via the KLF6 Tumor Suppressor during Induction of Myeloid Differentiation
Source: PLoS One. 2013 Aug 16;8(8):e71282. doi: 10.1371/journal.pone.0071282 (PMC3745464; doi:10.1371/journal.pone.0071282)

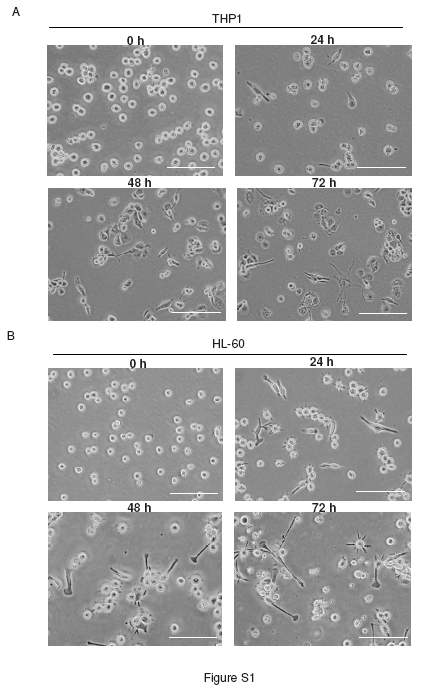

Supplement: Figure S1 — PMA induces cell differentiation in THP1 and HL-60 cells. THP1 or HL-60 cells were seeded on 6-well plates in RPMI containing 10% fetal bovine serum and treated with PMA (200 nM) for 24, 48 and 72 h. Cell morphology was observed under a phase-contrast microscope and photographed by a digital camera. Scale bar, 200 µm. (TIF) [file pone.0071282.s001.tif]

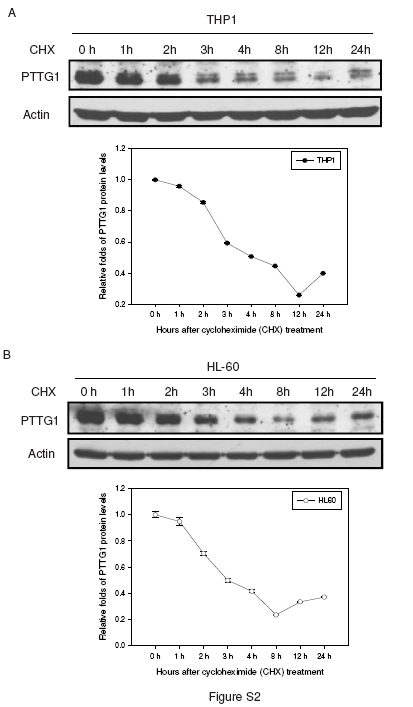

Supplement: Figure S2 — Measurement of the half-life of PTTG1 proteins in THP1 and HL-60 cells. THP1 or HL-60 cells (1×10^6^/well) were seeded on 6-well plates in RPMI containing 10% fetal bovine serum and treated with cycloheximide (CHX) (50 µg/ml) for indicated periods. The PTTG1 and actin proteins from total cellular lysate were detected by Western blot analysis in THP1 (A) and HL-60 (B). The immunoblot experiments were replicated at least three times, and a representative blot is shown. Relative fold of PTTG1 (normalized intensity of PTTG1 versus actin) is presented as the mean ± SD from three independent experiments and compared with 0 h group. (TIF) [file pone.0071282.s002.tif]

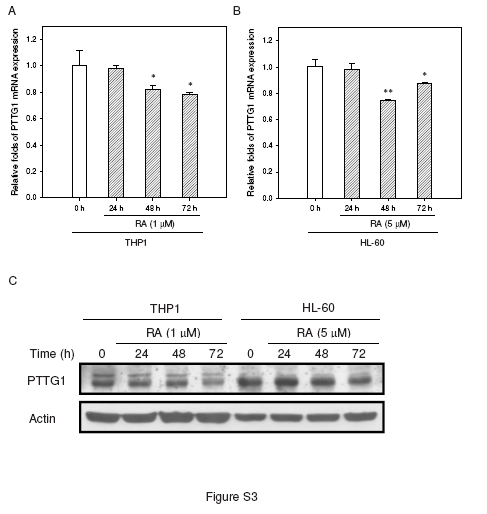

Supplement: Figure S3 — PTTG1 expression is down-regulated during retinoic acid (RA)-induced cell differentiation. Total cellular RNA was extracted and PTTG1 mRNA expression was determined by qRT-PCR in (A) THP1 and (B) HL-60 cells treated with retinoic acid (RA; 1 µM for THP1 and 5 µM for HL60) for indicated periods. Data represent the mean ± SD from five independent experiments. *p<0.05 and **p<0.01 represents significant differences compared with the 0 h group. (C) Western blot analysis was performed to detect PTTG1 and actin proteins in THP1 and HL-60 cells. The immunoblot experiments were replicated at least three times, and a representative blot is shown. (TIF) [file pone.0071282.s003.tif]

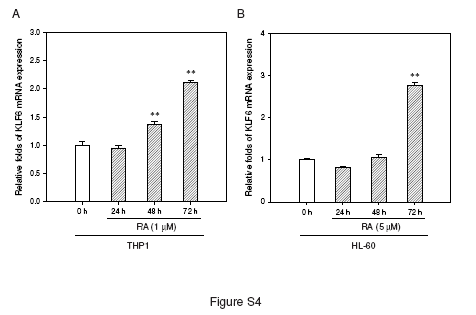

Supplement: Figure S4 — KLF6 expression is up-regulated during retinoic acid (RA)-induced cell differentiation. Total cellular RNA was extracted and KLF6 mRNA expression was determined by qRT-PCR in (A) THP1 and (B) HL-60 cells treated with retinoic acid (RA; 1 µM for THP1 and 5 µM for HL60) for indicated periods. Data represent the mean ± SD from five independent experiments. **p<0.01 represents significant differences compared with the 0 h group. (TIF) [file pone.0071282.s004.tif]

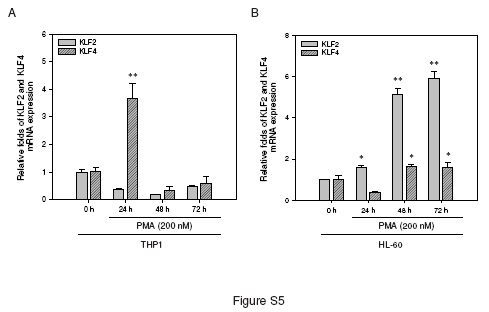

Supplement: Figure S5 — Examination of KLF2 and KLF4 mRNA expression levels during PMA-induced differentiation in THP1 and HL-60 cells. THP1 or HL-60 cells (1×10^6^/well) were treated with PMA (200 nM) for indicated periods. Total cellular RNA was extracted and KLF2 or KLF4 mRNA expression was determined by qRT-PCR in (A) THP1 and (B) HL-60 cells. Data represent the mean ± SD from five independent experiments. *p<0.05 and **p<0.01 represents significant differences compared with the 0 h group. (TIF) [file pone.0071282.s005.tif]

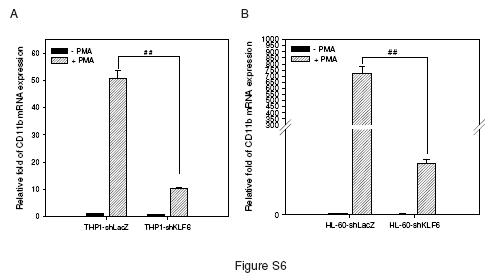

Supplement: Figure S6 — The effect of KLF6 knockdown on the CD11b mRNA expression during PMA-induced cell differentiation. Stable shLacZ or shKLF6 knockdown clonal lines were treated with PMA (200 nM) for 72 h. Total cellular RNA was extracted and CD11b mRNA expression was determined by qRT-PCR in (A) THP1 knockdown cells and (B) HL-60 knockdown cells. Data represent the mean ± SD from three independent experiments. ## p<0.01 represents significant differences compared with the PMA-treated shLacZ knockdown control group. (TIF) [file pone.0071282.s006.tif]

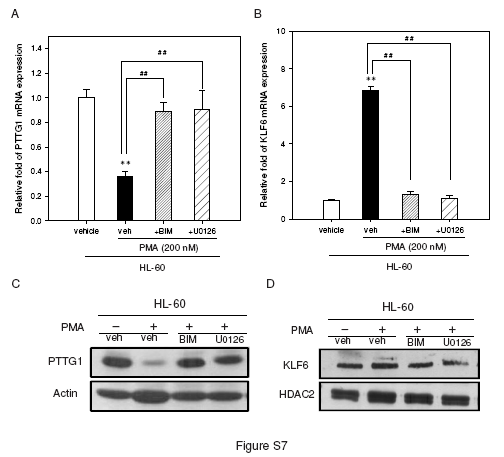

Supplement: Figure S7 — PKC and ERK signaling pathways are involved in the down-regulation of PTTG1 during PMA-induced HL-60 cell differentiation. HL-60 cells were pretreated with vehicle (veh, 0.1% DMSO), bisindolylmaleimide I (BIM, 5 µM) or U0126 (10 µM) for 30 min followed by exposure to PMA (200 nM) for 48 h. (A) Total cellular RNA was extracted and PTTG1 mRNA expression was determined by qRT-PCR. (B) Total cellular RNA was extracted and KLF6 mRNA expression was determined by qRT-PCR. Data represent the mean ± SD from three independent experiments. ** p<0.01 represents significant differences compared with PMA-non-treated cells. ## p<0.01 represents significant differences compared with the vehicle group. (C) The PTTG1 protein levels from total cellular lysates were detected by Western blot analysis. (D) The levels of KLF6 protein from nuclear extracts were determined by Western blot analysis. The immunoblot experiments were replicated at least three times, and a representative blot is shown. (TIF) [file pone.0071282.s007.tif]
